# Invasive and Noninvasive Intracranial Pressure Pulse Waveform in Neurocritical Care Patients with Different Cranium Integrity

**DOI:** 10.1007/s12028-025-02382-2

**Published:** 2025-10-01

**Authors:** Magdalena Kasprowicz, Agnieszka Kazimierska, Marta Hendler, Danilo Cardim, Zofia Czosnyka, Marek Czosnyka, Wellingson Paiva, Sergio Brasil

**Affiliations:** 1 Neuroengineering Lab (www.brainlab.pwr.edu.pl), Department of Biomedical Engineering, Wroclaw University of Science and Technology, Wybrzeze Wyspianskiego 27, 50-370 Wrocław, Poland; 2 brain4care, Johns Creek, GA USA; 3 Division of Neurosurgery, Department of Clinical Neurosciences, Addenbrooke’s Hospital, University of Cambridge, Cambridge, UK; 4 Division of Neurosurgery, Department of Neurology, University of São Paulo Medical School, São Paulo, Brazil; 5 Institute of Electronic Systems, Warsaw University of Technology, Warsaw, Poland

**Keywords:** Intracranial pressure, Pulse waveforms, Morphological analysis, Cerebrospinal compliance, Noninvasive monitoring

## Abstract

**Background:**

Pulse shape index (PSI) is a novel artificial intelligence–supported parameter that evaluates the pressure–volume compensatory reserve of the craniospinal system through intracranial pressure (ICP) pulse waveform classification. This study assessed the agreement between PSI derived from invasive ICP monitoring (PSI_ICP_) and noninvasive brain4care (B4C) sensor signal (PSI_B4C_) and investigated the influence of cranial integrity, age, and internal jugular vein (IJV) compression on PSI values.

**Methods:**

Simultaneous ICP and B4C monitoring was performed in 47 adult patients ( age: 43 (30) years) before and during IJV compression. Patients were grouped by cranial integrity: intact skull bone (*n* = 17), large skull fractures or craniotomies (*n* = 17), and craniectomies (*n* = 13). Pulse waveforms were automatically classified into four classes (from 1 = normal to 4 = pathological) by a neural network, and PSI was calculated as the weighted average of class numbers. Values are presented as median (interquartile range).

**Results:**

Bland–Altman analysis demonstrated good agreement between PSI_ICP_ and PSI_B4C_, with approximately 6% outliers. PSI was significantly higher in patients who underwent craniectomy compared with those with intact skulls (PSI_ICP_: 3.5 (0.8) vs. 2.0 (1.2) arbitrary units, *p* < 0.002; PSI_B4C_: 3.0 (0.4) vs. 2.0 (0.6) arbitrary units, *p* < 0.005) or those with craniotomies or large fractures (PSI_ICP_: 3.5 (0.8) vs. 2.0 (2.1) arbitrary units, *p* < 0.05; PSI_B4C_: 3.0 (0.4) vs. 2.0 (2.2) arbitrary units, *p* < 0.05). IJV compression did not affect PSI. Both PSI_ICP_ (r_s_ = 0.35, *p* < 0.02) and PSI_B4C_ (r_s_ = 0.37, *p* = 0.01) correlated with age.

**Conclusions:**

This study supports the B4C signal’s capability to noninvasively reflect ICP waveform morphology via PSI, offering a promising monitoring alternative. PSI appears to be influenced by age and craniectomy but not by a slight, sudden ICP change induced by IJV compression.

**Supplementary Information:**

The online version contains supplementary material available at 10.1007/s12028-025-02382-2.

## Introduction

The morphology of the intracranial pressure (ICP) pulse waveform reflects the dynamic interplay between arterial pulsatile blood inflow, venous outflow, and cerebrospinal pressure–volume compensatory mechanisms. During each cardiac cycle, arterial blood inflow and subsequent venous outflow induce transient changes in intracranial blood volume, leading to rhythmic fluctuations in ICP synchronized with heart rate. Results of previous studies have demonstrated that morphological analysis of ICP pulse waveforms is useful for continuous assessment of cerebrospinal pressure–volume compensatory reserve in patients in neurocritical care [1–5].

According to classical observations [2], a typical ICP pulse waveform consists of three main peaks: P1 (percussion wave), P2 (tidal wave), and P3 (dicrotic wave). In a normal waveform, P1—which reflects transmission of arterial pulsation through elastic walls of cerebral arteries—is the highest peak. It is followed by a smaller P2 peak (secondary to pulsatile arterial blood transport) and an even smaller P3. However, in scenarios of reduced compensatory reserve, such as increased intracranial volume due to cerebral edema or hemorrhage, P2 may become more prominent, indicating impaired compensatory mechanisms [6]. In pathological cases, when compensatory reserve is severely compromised, the ICP pulse waveform can exhibit a triangular or sinusoid-like shape characterized by a single dominant peak and the absence of clearly distinguishable secondary peaks.

Typical measures used in the study of ICP pulse morphology include the ratio of ICP peak height (the P2/P1 ratio) as well as peak occurrence times or time to maximum peak (TTP), which represent the time interval from the onset of the pulse to individual peaks or the most dominant peak, respectively [2, 7–9]. However, this approach requires precise identification of the peaks, which remains challenging because of the high variability in peak configuration over time and between patients [10]. Moreover, in cases of pathologically altered waveforms, peak localization becomes difficult, preventing the estimation of morphological metrics. The multitude of morphological parameters and their interdependencies further complicate straightforward clinical interpretation.

To address these limitations, a more generalized approach has been proposed, leveraging artificial intelligence to classify ICP pulse waveforms into four main shape categories according to P1, P2, and P3 configuration [11]. This method has been further developed in our recent study [12], leading to fully automated real-time shape classification without the need for precise peak localization, with simultaneous detection of artifacts and identification of invalid pulses for subsequent removal. Based solely on valid, artifact-free fragments of ICP recordings, this approach facilitates the computation of the pulse shape index (PSI), which allows for continuous tracking of changes in ICP pulse morphology. Previous studies have demonstrated the utility of PSI in early detection of intracranial hypertension [13], its association with treatment outcomes in patients with traumatic brain injury [14, 15], and correlation with the results of computed tomography scans [16] and patients’ age [17]. Nevertheless, despite its promising applications, a notable limitation of this approach is the invasive nature of ICP measurement.

The recently developed “brain4care” (B4C) system is an emerging noninvasive technique that detects pulsatile skull expansions occurring with each cardiac cycle and enables the acquisition of ICP-surrogate pulse waveform [18]. A comparison between traditional morphological metrics—the P2/P1 ratio and TTP—derived from standard invasive ICP pulse waveform and waveforms obtained using the B4C sensor has been conducted in clinical settings, demonstrating agreement between these two methods [19]. However, it remains to be demonstrated whether a more generalized approach based on classification of entire pulse wave shapes would yield similar agreement.

The primary aim of this study was to compare PSI derived from an invasive ICP waveform and noninvasive B4C pulsatile recordings in a group of patients in neurocritical care. A secondary objective was to investigate the impact of cranial integrity and aging on PSI values obtained from both ICP and B4C signal monitoring. Additionally, the study aimed to assess whether the ICP increase following internal jugular vein (IJV) compression has a comparable effect on PSI measurements obtained through invasive and noninvasive methods.

## Methods

### Ethical Approval/Informed Consent

This single-center study was conducted across six intensive care units at the Hospital das Clínicas São Paulo University in Brazil. The study protocol was approved by the local ethics committee and registered under the identifier NCT03144219 (ClinicalTrials.gov). All procedures adhered to relevant guidelines and regulations, and informed consent was obtained from legally authorized representatives or the next of kin due to the severity of the patients’ conditions.

### Data Acquisition and Study Protocol

**Fig. 1:**
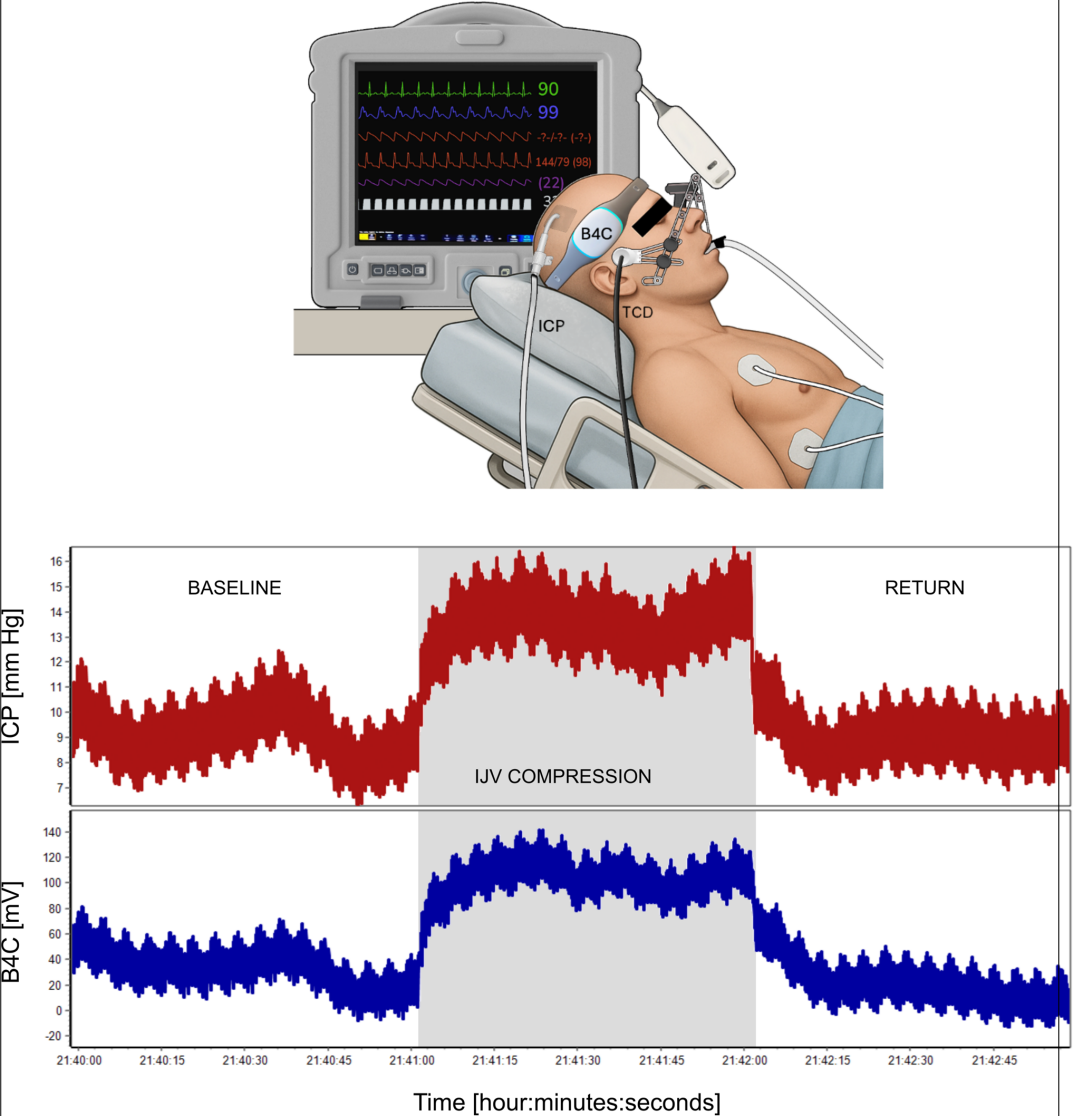
Schematic representation of the data acquisition setup (upper panel). A sample recording of invasive ICP and noninvasive B4C signals before, during and after IJV compression (lower panel). The compression period is highlighted by a grey-shaded area. Note that as part of our protocol, bilateral cerebral blood flow velocity in middle cerebral arteries was also recorded using TCD but was not analyzed in the present study. B4C, brain4care, ICP, invasive intracranial pressure, IJV, internal jugular vein, TCD, transcranial Doppler

Intraventricular ICP monitoring was performed using the Neurovent System, a catheter-tip microchip technology based on micro-electromechanical systems (Raumedic, Munchberg, Germany). At the same time, the B4C system (brain4care, São Carlos, São Paulo, Brazil)—a wearable device approved by the Food and Drug Administration (clearance number K201989)—was used to capture pulsatile micrometric skull expansions. The operational principles of this device have been described in a previous publication [18]. In brief, the system employs a highly sensitive displacement sensor placed in contact with the skin overlying the skull, which detects the pulsatile cranial expansions caused by ICP fluctuations occurring with each cardiac cycle. The B4C sensor was positioned in the frontotemporal region. The correctness of sensor placement was monitored by the responsible investigator (S.B.) to prevent any positioning errors that could degrade signal quality. Data obtained from the B4C sensor were used exclusively for research and did not influence clinical decision-making. Unlike invasive ICP sensors, the B4C system does not provide absolute values but only pulsatile changes; therefore, in this study, we focused on the morphology of the ICP and B4C pulse waveforms, using the average ICP level as a reference. A schematic representation of the data acquisition setup is presented in Fig. 1 (upper panel). The signals were recorded with a sampling frequency of 200 Hz using a custom data collection system (brain4care). A single three-minute recording session was conducted for each patient, during which ICP and B4C pulse waveforms were simultaneously recorded. During the monitoring, the patient remained in a supine position with the head elevated at 30°. After approximately one minute of baseline recording, ultrasound-guided manual compression of the IJV was applied for 60 s. This brief intervention was intended to minimize significant changes in systemic parameters while allowing for the assessment of subtle variations in ICP values and waveform characteristics. This maneuver induced a plateau wave in both recordings (see Fig. 1, lower panel).

### Participants

The original data set comprised 74 patients in neurocritical care with either traumatic or nontraumatic acute brain injury who required ventilatory support and in whom continuous ICP and B4C waveform recording was performed. The selection criteria are detailed in the flowchart in Suppl. Figure 1. Only high-quality ICP and B4C signals—with pulse waveforms classified as valid by a deep learning model (please refer to the “Signal preprocessing” section for details)—recorded in adult patients (age > 18 years) were included in further analyses.

Patients were stratified into three groups based on cranial status: (1) those with an intact skull, (2) those with large skull fractures or who had undergone craniotomies, and (3) those who had received craniectomies. A detailed summary of the studied sample is presented in the “Results” section.

### Signal Preprocessing

**Fig. 2:**
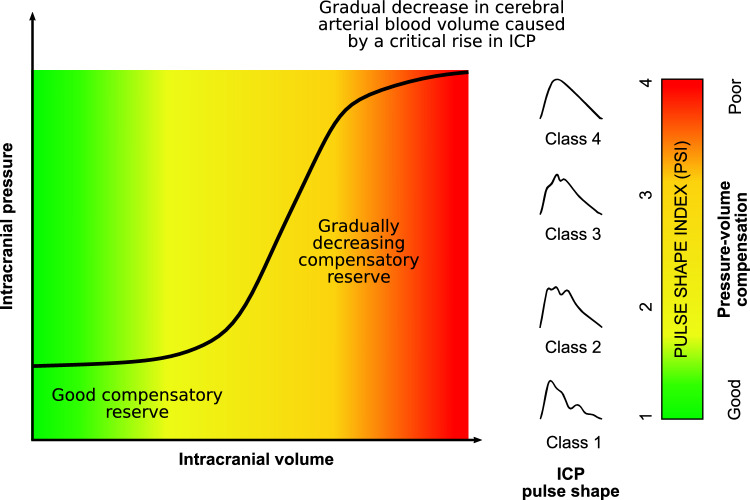
Interpretation of the intracranial pressure (ICP) pulse shape index (PSI). Low PSI values indicate normal ICP pulse waveform shape and good cerebrospinal compensatory reserve (green zone). As the compensatory reserve gradually decreases, the ICP pulse waveform shape becomes progressively altered (yellow–red zone). When the compensatory reserve is completely depleted, cerebrovascular reactivity is impaired, and a pathological ICP pulse waveform shape appears (red zone) (Color figure online)

A previously developed automated, artificial intelligence–based method [12] was employed for the classification of different ICP and B4C pulse waveform shapes as well as for artifact detection. Briefly, the algorithm comprises the following steps: segmentation of both signal recordings into individual pulse waveforms using a modified version of the Scholkmann algorithm [20]; normalization of the extracted pulse waveforms to a 0–1 range to eliminate the influence of pulse amplitude; resampling of the pulse waveforms to a uniform length of 180 samples, thereby reducing the influence of heart rate variability; and classification of ICP pulse waveform shapes using a residual neural network–based model [12]. As part of the morphological classification, the model distinguishes four waveform types (see Fig. 2): class 1 represents a normal waveform with a dominant peak P1; class 2 corresponds to a potentially pathological waveform characterized by increased prominence of peak P2, although P1 remains higher than P3; class 3 indicates a likely pathological waveform in which both P2 and P3 are prominent and dominate over P1; and class 4 reflects a pathological waveform with a rounded or triangular shape displaying only a single visible peak. Additionally, the model identifies distorted waveforms or errors in pulse detection, which are marked as artifacts and subsequently excluded to ensure that only valid signal segments are retained for further analysis.

### Pulse Shape Index

The model’s classification results (after exclusion of artifacts) were used to calculate PSI within moving ten-second windows (no overlap). PSI is defined as the weighted sum of class numbers (*i*) multiplied by the fraction of pulses in each class (*p*_*i*_), according to the formula:

1 $${\mathrm{PSI}} = \mathop \sum \limits_{i = 1}^{4} i \cdot p_{i}$$

Thus, PSI reflects the average class number over a given period, continuously capturing changes in pulse waveform shape on a scale from 1 (exclusively normal waveforms of class 1) to 4 (exclusively pathological waveforms of class 4), as illustrated in Fig. 2. PSIs calculated from ICP and B4C signals were respectively labeled as PSI_ICP_ and PSI_B4C_.

### Statistical Analysis

The normality of data distributions was assessed using the Shapiro–Wilk test. Because of the small sample size and the fact that the hypothesis of normality was rejected for most of the analyzed variables, nonparametric tests were applied.

The effect of cranial integrity (intact skull, large skull fractures or craniotomies, and craniectomies) on PSI was assessed using the Kruskal–Wallis test with post hoc comparisons (Dunn’s test). The results were presented as follows: H_K-W_(df, n) = H-statistic , *p* value, wherein df denotes degrees of freedom, *n* denotes the number of patients. Differences in PSI values obtained from ICP and B4C, both before and during IJV compression, were compared using the Wilcoxon signed-rank test. The correlations between PSI_ICP_ and PSI_B4C_ as well as their relationship with age were determined using Spearman’s rank correlation coefficient. To test the main effects of age (categorized into two groups: ≤ 43 years and > 43 years based on the median age of the analyzed cohort) and cranial condition as well as their interactions’ effects on PSI_ICP_ and PSI_B4C_, the Scheirer–Ray–Hare test was used. The results were presented as follows: H_S-R-H_(df effect, df error) = H-statistic, *p* value, wherein df denotes degrees of freedom. The effect size was assessed using partial η^2^ and expressed in percentage. The agreement between PSI_ICP_ and PSI_B4C_ was assessed using Lin’s concordance correlation coefficient (CCC) and Bland–Altman analysis, with separate analyses performed for each cranial condition both before and during IJV compression.

Statistical significance was set at *p* < 0.05. Data are presented as medians with interquartile ranges (IQRs) unless indicated otherwise. Statistical analyses were performed using Statistica software (v13, Tibco, Palo Alto, CA, USA) and Python 3.7.

## Results

### Patient Characteristics

Out of the initial dataset of 74 patients, 22 were excluded due to poor signal quality—of which 20 (91%) were rejected due to inadequate invasive ICP recordings—and 5 were excluded for being younger than 18 years of age. This resulted in a final cohort of 47 patients included in the analysis. In all 47 patients, IJV compression was successfully performed for 60 s without interruption. Their clinical characteristics are summarized in Table 1.

**Table 1:** Clinical characteristics of the final patient sample. Data are presented as median (interquartile range) or as number of occurrences (with optional percentage of the whole group)

Variable	
Age	43 (30) years
Male sex	31 (66%)
Pathology	
Traumatic brain injury	34
Marshall Score	
2	17 (50%)
3	2 (6%)
SEM	15 (44%)
Subarachnoid hemorrhage	7
Modified Fisher	
3	1 (14%)
4	6 (86%)
Neurosurgery	
No	17 (36%)
Craniotomy	17 (36%)
Craniectomy	13 (28%)
Sedation regimen	
No sedation	6 (13%)
Dexmedetomidine/fentanyl	8 (17%)
Propofol/fentanyl	23 (49%)
Propofol/midazolam/fentanyl	9 (19%)
Thiopental/fentanyl	1 (2%)
SAPS3	60 (12)
Admission GCS	6.0 (4.6)
In-hospital mortality	14 (30%)

*GCS* Glasgow Coma Scale, *SAPS3* Simplified Acute Physiologic Score III, *SEM* Surgically Evacuated Mass

### Agreement Between Invasive and Noninvasive PSI

**Fig. 3:**
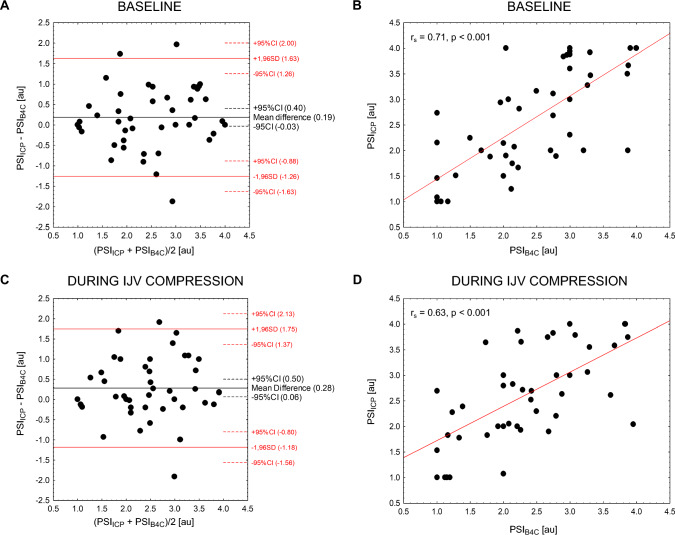
Bland–Altman plots (left column) and correlations (right column) between invasive and noninvasive PSIs for pooled data (*n* = 47) before (upper panel) and during (lower panel) IJV compression. au, arbitrary units, B4C, brain4care, CI, confidence interval, ICP, intracranial pressure, IJV, internal jugular vein, PSI, pulse shape index, PSI_B4C_, PSI derived from noninvasive B4C measurement, PSI_ICP_, PSI derived from invasive ICP measurement

Illustrative examples of ICP and B4C pulse waveforms with matching shapes (belonging to the same pulse shape class) as well as those differing in shape are presented in Suppl. Figure 2. Invasive PSI_ICP_ and noninvasive PSI_B4C_ were significantly correlated both before and during IJV compression (see Fig. 3, right panel). The pooled-data Bland–Altman analysis demonstrated good agreement between PSI derived from ICP and the B4C signal, with approximately 6% outliers and small mean differences (biases) between the two methods both before and during compression (see Fig. 3, left panel). Lin’s CCC was 0.71 at baseline and 0.60 during compression, indicating good and moderate agreements between invasive and noninvasive PSI, respectively. The correlation plots between PSI_ICP_ and PSI_B4C_ accounting for patients’ cranium integrity, along with the Bland–Altman plots, are presented in Suppl. Figure 3 and 4.

### Impact of Cranium Condition on PSI

**Fig. 4:**
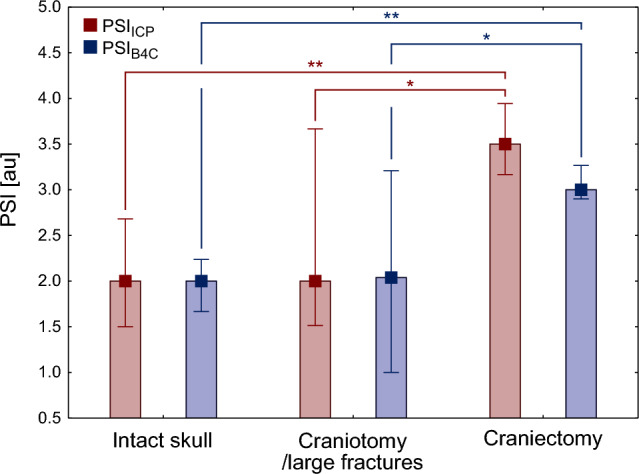
Impact of cranium integrity on PSI_ICP_ and PSI_B4C_ at baseline. The values are presented as medians (squares) and interquartile ranges (whiskers). ** denotes *p*_post hoc_ < 0.01, * denotes *p*_post hoc_ < 0.05. au, arbitrary units, B4C, brain4care, ICP, intracranial pressure, PSI, pulse shape index, PSI_B4C_, PSI derived from noninvasive B4C measurement, PSI_ICP_, PSI derived from invasive ICP measurement

When compared across the three groups, there were significant differences in both PSI_ICP_ and PSI_B4C_ between patients with different status of cranium integrity (PSI_ICP_: H_K-W_ (2, 47) = 11.97, *p* = 0.0025; PSI_B4C_: H_K-W_ (2, 47) = 10.72, *p* = 0.0047). Both PSI_ICP_ and PSI_B4C_ were the highest in patients after craniectomy, with PSI_ICP_ being higher than PSI_B4C_, although this difference did not reach statistical significance (*p* = 0.05) (see Fig. 4). There were no differences in PSI (either derived from ICP or B4C) between patients with intact skull and those with craniotomy or large fractures.

In patients after craniectomy, mean ICP was elevated (16.7 (2.8) mm Hg), but the differences in ICP level between the three cranium conditions did not reach statistical significance (H_K-W_(2, 47) = 5.91, *p* = 0.05) (see Suppl. Figure 5).

### Impact of Age on PSI

**Fig. 5:**
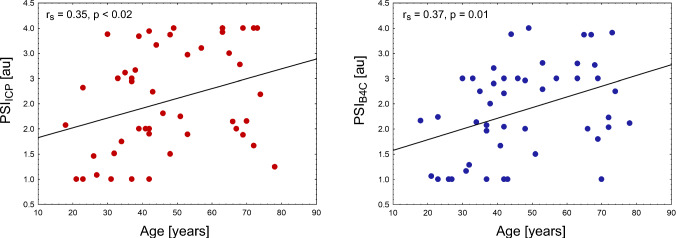
Relationship between age and PSI derived from invasive (left panel) and noninvasive (right panel) measurement at baseline. au, arbitrary units, B4C, brain4care, ICP, intracranial pressure, PSI, pulse shape index, PSI_B4C_, PSI derived from noninvasive B4C measurement, PSI_ICP_, PSI derived from invasive ICP measurement

There were no significant differences in age between the groups of patients with different cranial conditions (H_K-W_(2, 47) = 0.21, *p* = 0.90): median age of patients with intact skull was 49 (29) years; for large skull fractures or craniotomies, median age was 42 (26) years, and for craniectomies, median age was 42 (29) years. Pooled-data analysis demonstrated a moderate correlation between age and both PSI derived from ICP (r_s_ = 0.35, *p* < 0.02) and PSI derived from B4C (r_s_ = 0.37, *p* = 0.01) recorded at baseline (see Fig. 5).

### Interaction Effect of Age and Cranium Integrity on PSI

#### *InvasivePSI*_*ICP*_

**Fig. 6:**
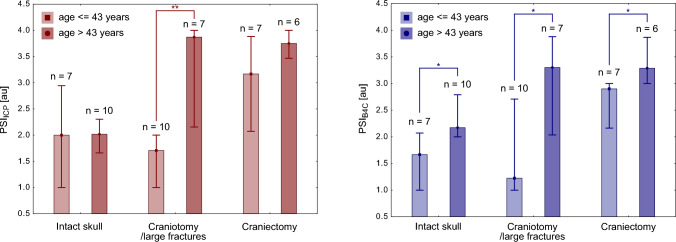
Interaction effect of cranium condition and age on PSI derived from invasive PSI_ICP_ (left panel) and noninvasive PSI_B4C_ (right panel). The values are presented as medians (squares) and interquartile ranges (whiskers). ** denotes *p*_post hoc_ < 0.01, * denotes *p*_post hoc_ < 0.05. au, arbitrary units, B4C, brain4care, ICP, intracranial pressure, PSI, pulse shape index, PSI_B4C_, PSI derived from noninvasive B4C measurement, PSI_ICP_, PSI derived from invasive ICP measurement

The effects of cranium integrity and age on PSI_ICP_ were both statistically significant (cranium integrity: H_S-R-H_(2, 41) = 22.2, *p* < 0.0001; age: H_S-R-H_(1, 41) = 11.1, *p* < 0.001). Cranium integrity had a stronger influence, accounting for 35% of the variance in PSI_ICP_, whereas age contributed 21%. Additionally, a significant interaction effect was observed (H_S-R-H_(2, 41) = 11.1, *p* < 0.004), suggesting that the impact of cranium condition on PSI_ICP_ varied depending on age. Specifically, PSI_ICP_ was elevated in patients who were older and had undergone craniotomies or craniectomies. However, among patients with an intact skull, there was no significant difference in PSI_ICP_ between younger and older individuals (see Fig. 6, right panel).

#### *Noninvasive PSI*_*B4C*_

For PSI_B4C_, both cranium integrity and age were also significant factors (cranium integrity: H_S-R-H_(2, 41) = 18.7, *p* < 0.0001; age: H_S-R-H_(1,41) = 16.1, *p* < 0.0001). The effect sizes were slightly different compared with PSI_ICP_, with cranium integrity explaining 31% of the variance and age accounting for 28%. However, in contrast to PSI_ICP_, there was no significant interaction effect (H_S-R-H_(2, 41) = 4.3, *p* = 0.11), indicating that the influence of age on PSI_B4C_ was consistent across different cranium conditions. Regardless of skull integrity, patients who were older (age > 43 years) exhibited higher PSI_B4C_ values (see Fig. 6, left panel).

### Impact of IJV Compression on PSI

**Fig. 7:**
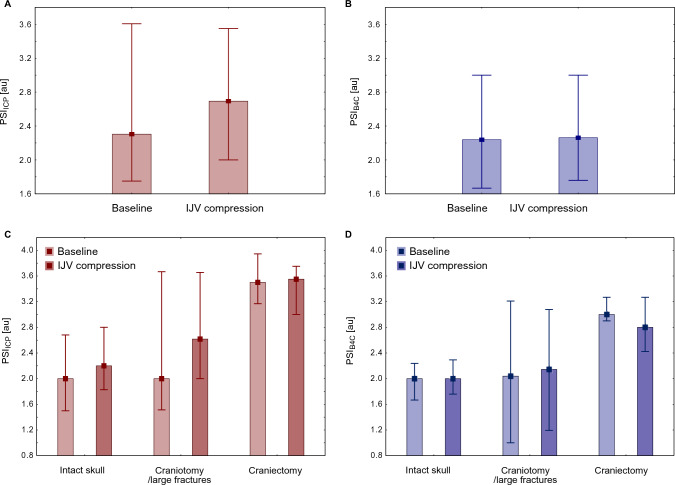
Impact of IJV compression on invasive PSI_ICP_ (left column) and noninvasive PSI_B4C_ (right column) for pooled data (*n* = 47, upper panel) and in groups of patients stratified by cranium condition (lower panel). No significant differences in either PSI_ICP_ or PSI_B4C_ between baseline and IJV compression were found regardless of cranium integrity. au, arbitrary units, B4C, brain4care, ICP, intracranial pressure, IJV, internal jugular vein, PSI, pulse shape index, PSI_B4C_, PSI derived from noninvasive B4C measurement, PSI_ICP_, PSI derived from invasive ICP measurement

IJV compression resulted in a significant increase in median ICP both for the pooled data (baseline 11.8 (8.3) mm Hg vs. IJV compression 17.1 (7.1) mm Hg, *p* < 0.001) and in separate cranium integrity subgroups (see Suppl. Figure 6). However, IJV compression did not cause statistically significant changes in PSI_ICP_ or PSI_B4C_, either when analyzing the entire group of patients or when stratifying by cranium condition (see Fig. 7).

## Discussion

The results of our study demonstrated that PSI derived from invasive catheter-based ICP measurements and the noninvasive B4C signal are correlated, with satisfactory agreement between the two methods. Similar findings were observed for conventional morphological metrics, such as the P2/P1 ratio and TTP, which rely on peak identification in pulse waveforms, using the same or overlapping cohort of patients [7, 19]. In this study, we confirmed that a more general approach—one that accounts for the entire waveform shape rather than relying on complex peak identification—shows technical feasibility and physiological relevance when applied to noninvasive B4C signals. Although our results are encouraging, they primarily support further investigation of this method rather than establish its suitability for routine continuous monitoring in clinical practice.

Despite the heterogeneity of the sample, which included patients with varying cranial integrity (intact skulls, skull fractures, and post-craniectomy cases) as well as different underlying brain pathologies (traumatic brain injury, subarachnoid hemorrhage, stroke), the pooled data still demonstrated a good correspondence between invasive and noninvasive pulse morphology. However, the strongest agreement between PSI_ICP_ and PSI_B4C_ was observed in patients with an intact skull. In this group, the mean difference between the two methods was close to zero, with all discrepancies lying within the limits of agreement. The greatest and positive mean difference between PSI_ICP_ and PSI_B4C_ was observed in patients who underwent craniectomy, suggesting that the noninvasive approach may underestimate PSI values in this subgroup [21]. Lin’s CCC was also the lowest in this group of patients, indicating moderate agreement between invasive and noninvasive PSI. Furthermore, in patients who underwent craniectomy, the correlation coefficient between PSI_ICP_ and PSI_B4C_ did not reach statistical significance.

Notably, PSI values—both invasive and noninvasive—were the highest in patients who underwent craniectomy compared with those with an intact skull or those presenting large fractures or postsurgical cranial modifications. This finding may appear counterintuitive, as skull decompression is expected to lead to a significant reduction in ICP and a corresponding increase in cerebrospinal compensatory reserve (indicated by lower PSI) [22]. In our study, ICP remained relatively high following craniectomy, but the values are consistent with those reported in previous research [23]. The elevated mean ICP in patients who underwent craniectomy could, in part, explain the higher PSI values observed in this group. Furthermore, the patients in this sample were assessed during the early days following severe acute brain injury. During this convalescence period, brain edema is significant and may have influenced our results, as previously observed by this group using a different approach [19]. A longer-term study including patients who underwent craniectomy could provide further clarification on this issue.

Nevertheless, prior studies have suggested that ICP itself has a relatively minor influence on ICP pulse morphology, and other factors—such as the patient’s age—may play a more significant role [17, 24–26]. It should be noted that patients who underwent craniectomy typically present with more severe clinical conditions that may also result in decreased compensatory reserve.

PSI values derived from both ICP and B4C measurements were consistently higher in patients who were older. There were no significant age differences between the three patient groups stratified by cranial integrity. However, when analyzing the combined effects of cranial condition and age, we found that the age-related differences in PSI were more pronounced in patients with compromised skull integrity. In patients who were older, PSI_ICP_ was significantly higher in those with disrupted skull integrity, whereas this age-related difference was less pronounced in individuals with an intact skull. Earlier research on PSI_ICP_ in traumatic brain injury patients with an intact skull demonstrated a significant age-dependent relationship [17]. Therefore, the weaker age-related difference in PSI_ICP_ in patients with an intact skull observed in this study may be attributed to the relatively small sample size. Regarding PSI_B4C_, the values were consistently higher in older individuals regardless of cranial integrity, a trend also observed in healthy populations based on P2/P1 ratio analysis [27]. Nevertheless, cranial integrity appeared to exert a greater influence on PSI (both invasive and noninvasive) than age.

The association between PSI and age is most likely linked to cerebrovascular changes, as cerebral arteries become stiffer with age, leading to more pronounced pulsatile pressure [28]. Additionally, prior studies have suggested that pulse morphology is primarily determined by changes in cerebral blood volume [29–31]. Craniectomy significantly alters the pressure–volume dynamics within the intracranial space. The removal of bone may facilitate a more dominant influence of pulsatile blood volume fluctuations, leading to a predominance of peak P2 and a smoother, more sinusoidal ICP waveform.

Our findings demonstrate the association between increasing age and higher PSI values. However, the specific age at which interpretation of PSI should be adjusted or becomes critical remains to be determined. Establishing age-specific normative ranges and clinical thresholds will require larger, longitudinal studies. Further studies are essential to refine the clinical utility and accuracy of PSI-based monitoring across diverse patient populations, particularly as age-related vascular changes may affect interpretation of the measurements.

An earlier study using the P2/P1 ratio has demonstrated that the ICP increase induced by IJV compression leads to a reduction in this ratio in patients after craniectomy, whereas the opposite effect was observed in patients with an intact skull or those with altered skull integrity [7]. This observation suggested that after craniectomy, compensatory reserve is enhanced to protect the brain against adverse ICP elevation. However, in our study, neither PSI derived from ICP nor PSI derived from B4C confirmed this observation, although PSI_B4C_ exhibited a downward trend during IJV compression in patients who underwent craniectomy (without reaching statistical significance). One possible explanation for this discrepancy is that PSI may be less sensitive to subtle shifts in the position of peaks P1 and P2, classifying a broader range of peak configurations into a single category. Another potential explanation is that peak analysis becomes less accurate as the pulse shape becomes more rounded, with peak P1 gradually diminishing or even becoming undetectable, leading to potential inaccuracy of the results.

Nonetheless, in our study, IJV compression induced a significant increase in ICP but had no significant effect on PSI in any of the analyzed groups, regardless of cranial integrity status. In fact, the absence of changes in cerebrospinal compensatory reserve during IJV compression was expected. The compliance of the system is inversely related to the difference between ICP and pressure in the sagittal sinus [32, 33]. Because both pressures increase during IJV compression, the pressure gradient remains mostly unchanged, resulting in no alteration in compensatory reserve. Another explanation is that PSI remains relatively stable during gradual elevations in ICP, unless these changes are sufficient to alter the overall morphology of the waveform—typically closer to the point of pressure–volume decompensation. Importantly, cerebrospinal compliance may be reduced before ICP elevation occurs, particularly in older individuals or those with preexisting vascular pathology, resulting in elevated PSI values despite normal or borderline ICP levels. As such, PSI is not a standalone marker for rising ICP but may offer clinical value in identifying patients with an early decrease in compliance, even when mean ICP remains within normal limits. This makes it potentially valuable for identifying patients at risk of sudden ICP increase before clinical deterioration becomes apparent. Because PSI does not reflect absolute ICP values, it cannot replace invasive monitoring. Rather, it provides complementary information on cerebrospinal compensatory reserve and may support decision-making in settings where invasive tools are unavailable. Further validation studies are needed to determine the prognostic relevance and practical utility of noninvasive PSI in neurocritical care.

## Limitations

The primary limitation of this study is the relatively small sample size. This was partly caused by a high data exclusion rate due to poor quality of the invasive ICP signal, which accounted for 91% of the excluded recordings. The issue stemmed from the data acquisition setup, which produced low-resolution ICP signal in part of the recordings. After upgrading the monitoring configuration, the quality of the ICP signal improved sufficiently to enable accurate morphological analysis. Another limitation of the study is the lack of analysis regarding the effects of sedation, neuromuscular blockade, and ventilation parameters (e.g., positive end-expiratory pressure) on ICP and B4C waveform morphology. Although such factors may influence signal characteristics, detailed data were not uniformly available. All recordings were, however, obtained during clinically stable conditions following standard care protocols. IJV compression was performed only once per patient, without repeated measurements over time. Although multiple assessments could have provided more robust data on intraindividual variability, repeated interventions were avoided for ethical and practical reasons in this critically ill population. The morphological analysis of ICP and B4C pulse waveforms was previously conducted in the same clinical data set [7, 19], although the previous studies used a traditional approach that relies on challenging and often imprecise peak identification. Furthermore, the classification model for ICP pulse waveform morphology was trained exclusively on invasive ICP pulse waveform recordings. This may have introduced potential inaccuracies in our analysis, as the morphology of B4C pulsations may inherently differ due to variations in measurement location (intraventricular vs. extracranial) and measurement technique (pressure in mm Hg vs. skull expansion in micrometers). Although moderate ICP elevations were observed in some cases, the study cohort did not include patients with sustained intracranial hypertension. Future studies should specifically address this subgroup to better evaluate the clinical utility of noninvasive PSI monitoring. Finally, we did not analyze the amplitude of the ICP and B4C waveforms. Although the PSI metric was intentionally designed to focus solely on waveform shape, pulse amplitude may carry additional physiological information related to cerebrospinal compliance. Moreover, we did not evaluate how omitting amplitude normalization might affect PSI values. Future work may incorporate both shape and amplitude features to provide a more comprehensive assessment of cerebrospinal compliance.

## Conclusions

Our study demonstrated that the morphologies of invasive ICP and noninvasive B4C pulse waveforms correspond to each other in patients in neurocritical care. However, the results of waveform shape analysis in patients with compromised skull integrity—particularly after craniectomy—should be interpreted with caution, regardless of whether they are based on invasive or noninvasive measurements. Additionally, patient age should also be considered in morphological analysis.

## Supplementary Information

Below is the link to the electronic supplementary material.

Supplementary file1 (DOCX 3270 KB)

## Data Availability

The data that support the findings of this study are available upon reasonable request.
