# Supplementary material for: Invasive and Noninvasive Intracranial Pressure Pulse Waveform in Neurocritical Care Patients with Different Cranium Integrity
Source: Neurocrit Care. 2025 Oct 1;44(1):282–93. doi: 10.1007/s12028-025-02382-2 (PMC12819473; doi:10.1007/s12028-025-02382-2)
Supplement: Supplementary file 1 — Supplementary file1 (DOCX 3270 KB) [file 12028_2025_2382_MOESM1_ESM.docx]

**Supplementary material**

*Patient selection flow chart*


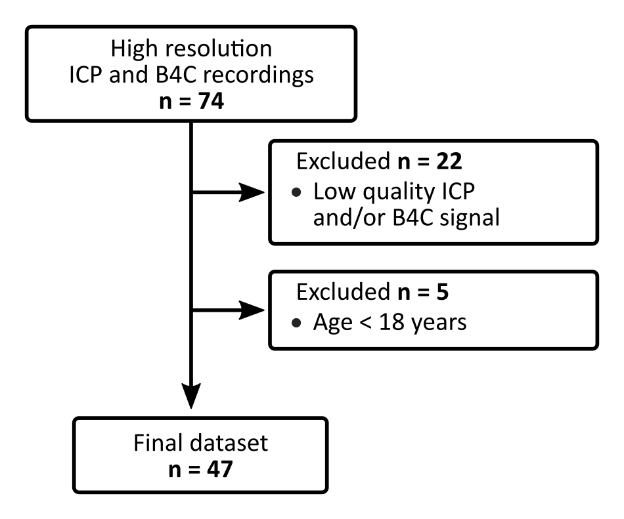


Suppl. Fig. 1. Selection criteria for the final patient dataset included in the study. ICP—intracranial pressure, B4C—Brain4care signal, n—number of patients.

*Examples of intracranial pressure (ICP) and Brain4care (B4C) pulse waveform morphology*


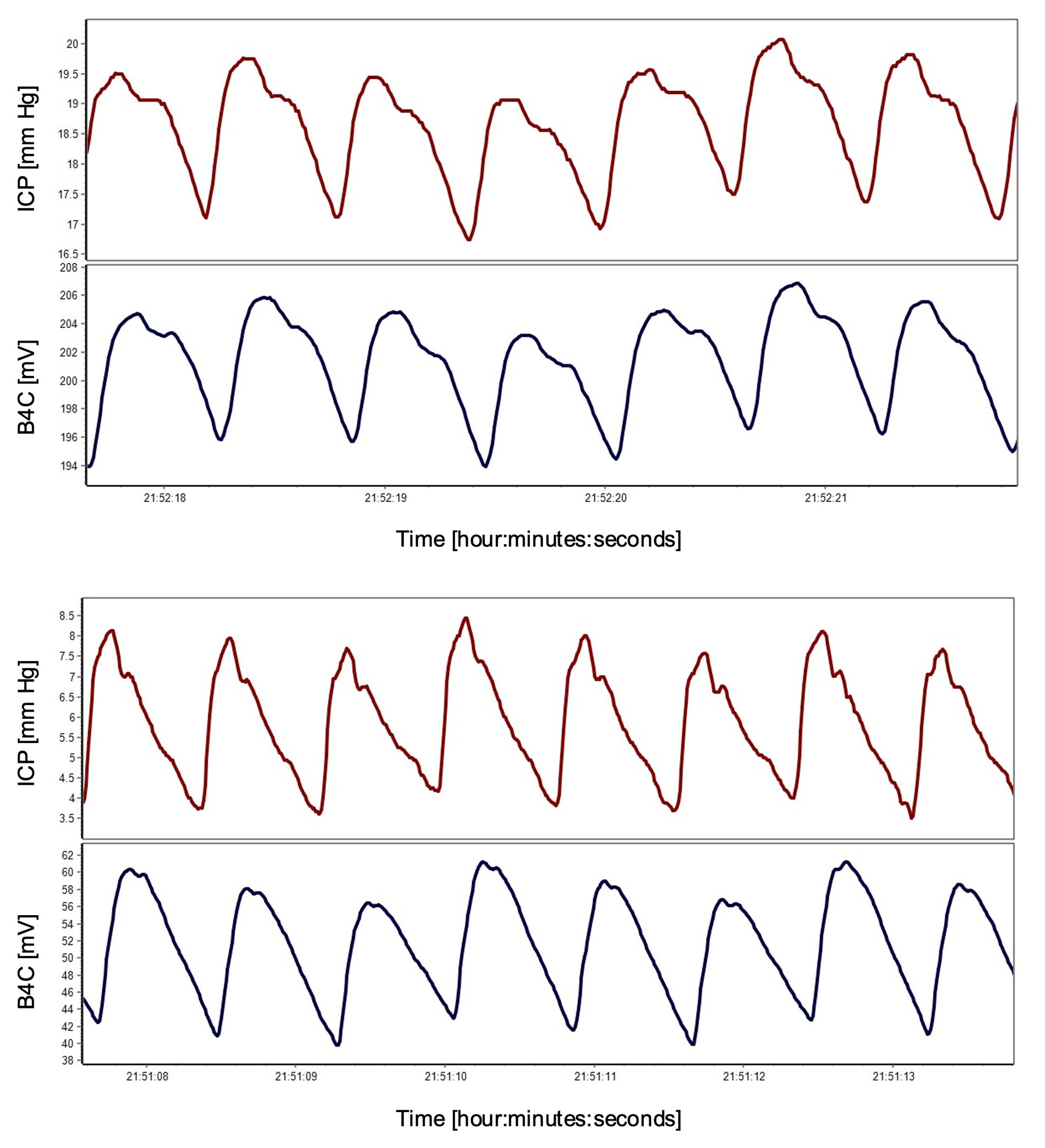


Suppl. Fig. 2. Illustrative examples of intracranial pressure (ICP) and brain4care (B4C) pulse waveforms with: matching shapes (i.e. belonging to the same pulse shape class) (*upper panel*), different shapes (*lower panel*).

*Bland–Altman analysis and Lin’s Concordance Correlation Coefficient* (CCC)

The Bland–Altman analysis demonstrated a good agreement between PSI derived from ICP and B4C, with relatively small mean differences (biases) between the two methods, both before and during IJV compression and regardless of the status of cranium integrity (see Suppl. Fig. 4). The Lin’s CCC values indicated moderate to good agreement between PSI_ICP_ and PSI_B4C_ for patients with intact skull or those presenting large fractures or post-surgical cranial modifications, both before and during IJV compression (intact skull: CCC_baseline_=0.64, CCC_compression_=0.48; craniotomy/large fractures: CCC_baseline_=0.63, CCC_compression_=0.58) while post-craniectomy patients showed moderate to weak agreement (CCC_baseline_=0.56, CCC_compression_=0.34).


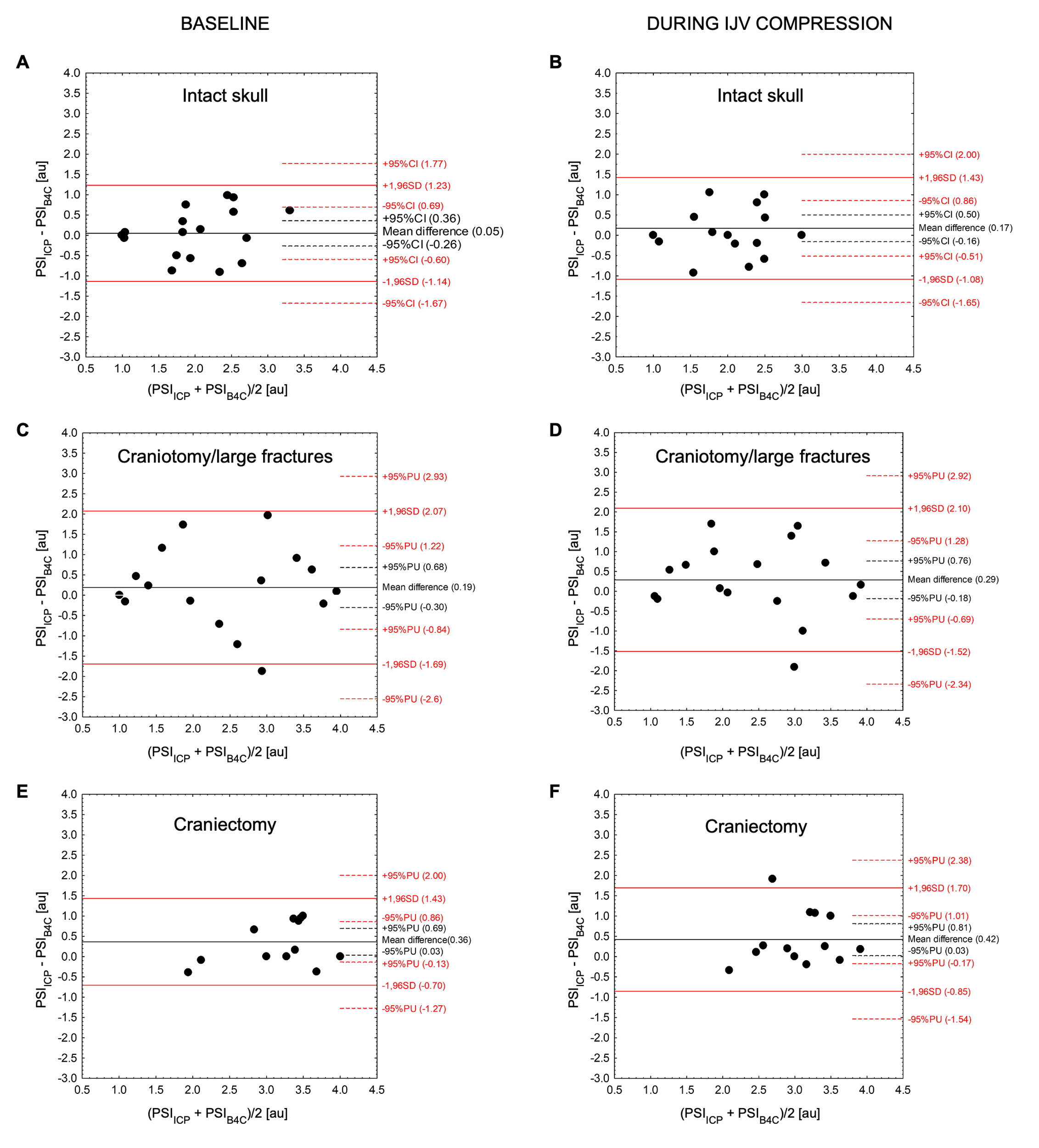


Suppl. Fig. 3. Bland–Altman plots for the invasive vs. non-invasive method of pulse shape index (PSI) estimation before (*left panel*) and during (*right panel*) internal jugular vein (IJV) compression in groups of patients stratified by cranium condition. PSI_ICP_—PSI derived from invasive intracranial pressure (ICP) measurement, PSI_B4C_—PSI derived from non-invasive brain4care (B4C) measurement, au—arbitrary units.

*Correlation analysis*

PSI derived from ICP (invasive) and B4C (non-invasive) was significantly correlated in patient groups with an intact skull and after craniotomy, both before and during IJV compression. However, this relationship was insignificant in patients after craniectomy (see Supp. Fig. 4).


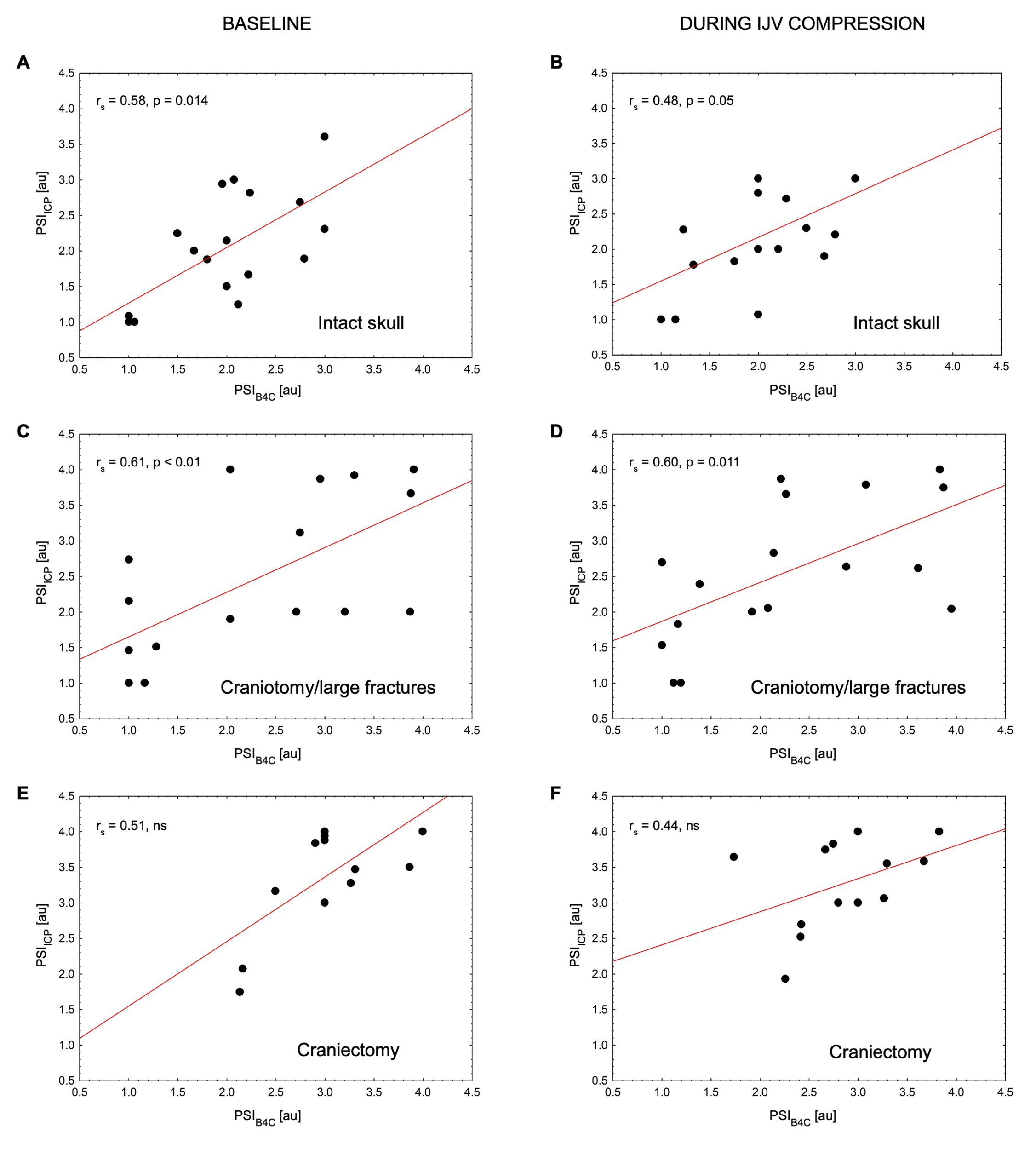


Suppl. Fig. 4. Correlations between invasive and non-invasive pulse shape index (PSI) before (*left panel*) and during (*right panel*) internal jugular vein (IJV) compression in groups of patients stratified by cranium condition. PSI_ICP_—PSI derived from invasive intracranial pressure (ICP) measurement, PSI_B4C_—PSI derived from non-invasive brain4care (B4C) measurement, au—arbitrary units, ns—result not statistically significant.

*Impact of cranium integrity on intracranial pressure*

In patients after craniectomy, ICP was elevated compared with patients with intact cranium and patients presenting large fractures or after craniotomy. However, the differences in ICP did not reach a statistically significant level (H(2, 47) = 5.91, *p* = 0.05)); see Suppl. Fig. 5.

*
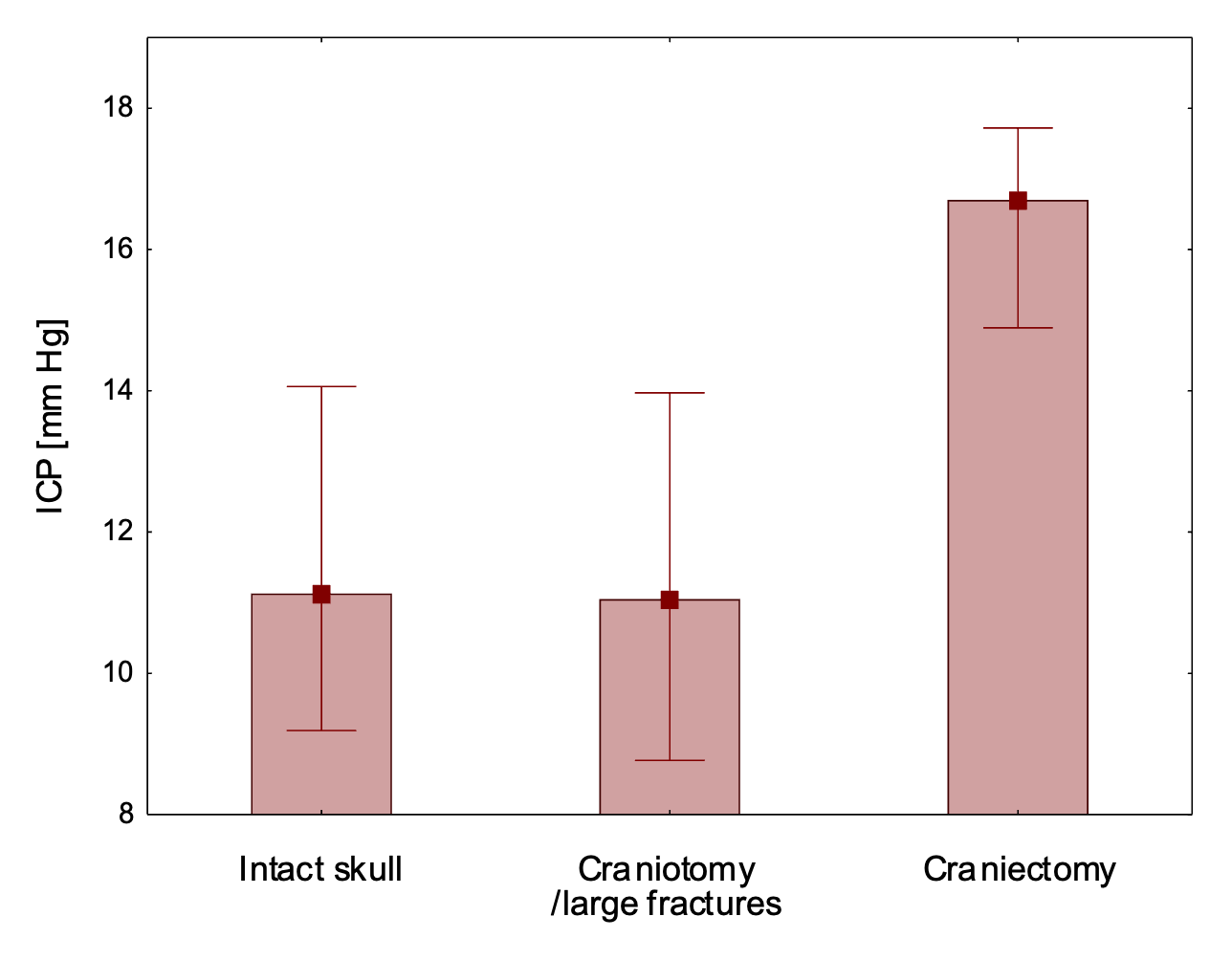
*

Suppl. Fig. 5. Impact of cranium integrity on intracranial pressure (ICP) level. The values are presented as medians (squares) and interquartile ranges (whiskers). The differences between the groups did not reach statistical significance.

*Impact of internal jugular vein compression on intracranial pressure*

IJV compression resulted in a significant increase in median ICP both for the pooled data and in separate cranium integrity subgroups (see Suppl. Fig. 6).


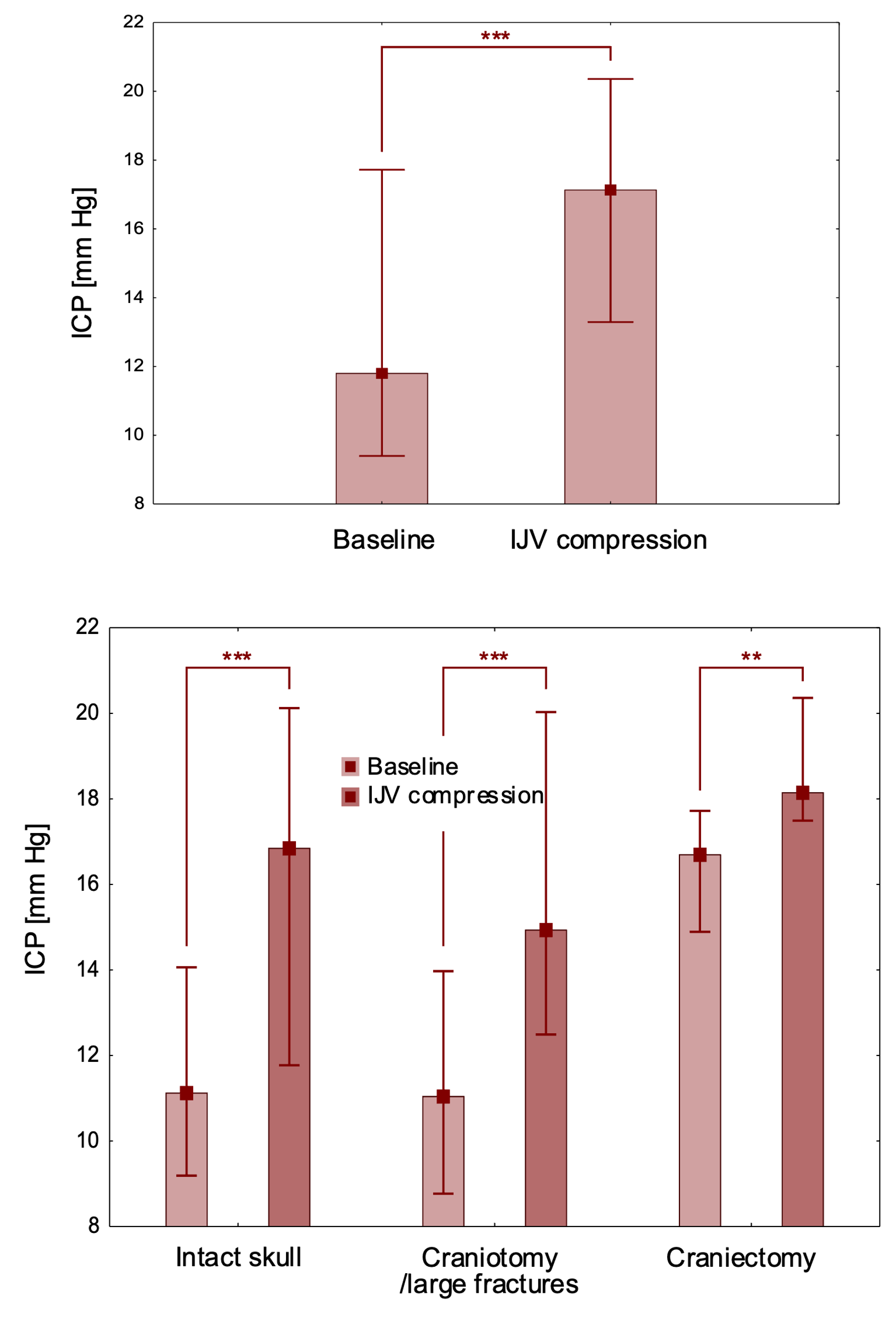


Suppl. Fig. 6. Effect of internal jugular vein (IJV) compression on intracranial pressure (ICP) in pooled data (n = 47) (*upper panel*) and in groups of patients stratified by cranium integrity (*lower panel*). The values are presented as medians (squares) and interquartile ranges (whiskers). *** denotes *p*_post-hoc_ < 0.001, ** denotes *p*_post-hoc_ < 0.01.
